# A New Pedicled Internal Mammary Osteomyocutaneous Chimeric Flap (PIMOC) for Salvage Head and Neck Reconstruction: Anatomic Study and Clinical Application

**DOI:** 10.1038/s41598-017-13428-7

**Published:** 2017-10-11

**Authors:** Guilherme C. Barreiro, Chelsea C. Snider, Flavio H. F. Galvão, Rachel R. Baptista, Kiril E. Kasai, Daniel M. dos Anjos, Marcus C. Ferreira

**Affiliations:** 1 Division of Plastic and Reconstructive Surgery, State University of Campinas, Campinas, SP 13080-888 Brazil; 2 Institute for Plastic Surgery, Southern Illinois University, Springfield, IL 62702 USA; 3 Department of Surgery, University of São Paulo, São Paulo, SP 05403-010 Brazil; 4 Division of Plastic Surgery, Syrian-Lebanese Hospital, São Paulo, SP 01308-050 Brazil

## Abstract

Well-vascularized composite tissue offers improved outcomes for complex head and neck reconstruction. Patients with vessel-depleted necks and failed reconstructions require alternative reconstructive options. We describe a pedicled internal mammary artery osteomyocutaneous chimeric flap (PIMOC) for salvage head and neck reconstruction. Bilateral dissections of 35 fresh cadavers were performed to study individual tissue components and vascular pedicles to develop the PIMOC technique. The flap was then utilized in a series of patients with vessel-depleted neck anatomy. The PIMOC was dissected bilaterally in all cadavers and there were no statistical differences in vascular pedicle caliber or length with regards to laterality or gender. Five patients subsequently underwent this procedure. The flaps included a vertical rectus abdominis myocutaneous component and a 6^th^ or 7^th^ rib with adjacent muscle and skin to restore bone defects, internal lining, and external coverage. All donor sites were closed primarily. There were no flap losses and all patients gained improvements in facial contour, speech and swallow. Although technically complex, the PIMOC is reproducible and provides a safe and reliable option for salvage head and neck reconstruction. The harvest of the 6^th^ or 7^th^ rib and rectus abdominis muscle renders an acceptable donor site.

## Introduction

Tertiary head and neck reconstruction has become more frequent with improvements in oncologic treatment^1,2^. The frozen, vessel-depleted neck is a challenge for the reconstructive surgery secondary to osteoradionecrosis, tissue fibrosis, volume loss, and contour deformity. Patients experience poor quality of life with chronic wounds and severe functional impairments, with few or no feasible reconstructive options^3–6^.

Although free tissue transfer is the gold standard for these complex head and neck reconstructions^7^, absent recipient vessels and scarce loco-regional flap options demand unusual techniques, some beyond customary free flap transfer. The literature describes complex procedures like distant vessel mobilization, vein grafts, AV loops, and distant pedicled flaps, with poor outcomes^8–13^.

As an alternative, the internal mammary vessels can serve as recipient for free tissue transfer or as pedicled axial flaps^14–18^. After anatomic cadaver study, we hypothesized that the internal mammary vessels could be used to develop an innovative pedicled flap for complex head and neck reconstruction.

This article describes research from the cadaver to the clinical setting for the application of a new Pedicled Internal Mammary Osteomyocutaneous Chimeric Flap (PIMOC) technique for head and neck reconstruction.

## Methods

### Anatomic study

Thirty-five fresh cadavers were used to study the anatomy of the neck, chest, and abdominal vessels to standardize the PIMOC technique. We designed individual tissue components, each supplied by their own vascular pedicle originating from a single internal mammary artery. The flap included the internal mammary artery (IMA), musculophrenic (MP) artery, 6^th^ and 7^th^ intercostal (IC) arteries, superficial superior epigastric perforator (SSEP), deep superior epigastric artery (DSEA), and deep inferior epigastric artery (DIEA). Vessel length and caliber were measured using a digital caliper. Photographic analysis was performed using the Image J software from the Research Services Branch, National Mental Health Institute, Bethesda, Maryland.

To harvest the PIMOC, we accessed the IMA pedicle through an inverted L sternotomy, with the superior transverse osteotomy at the level of the ipsilateral first intercostal space. We dissected the internal mammary pedicle from the posterior costosternal synchondroses and transversus thoracis muscle and ligated the second to fifth intercostal branches. We isolated the distal bifurcation of the internal mammary into the musculophrenic artery and the DSEA. The musculophrenic artery originates from the 6^th^ and 7^th^ intercostal arteries to supply the rib and the DSEA supplies the vertical rectus abdominis muscle flap (VRAM). In the upper abdomen, the SSEP from the deep superior epigastric vessels emerges from the anterior rectus fascia 4 to 8 cm from the xiphoid process, and supplies an ipsilateral oblique skin island that can be individually harvested and included in the flap^19,20^. Perforator position, skin island orientation and dimensions were observed with intra-arterial dye studies.

**Figure 1:**
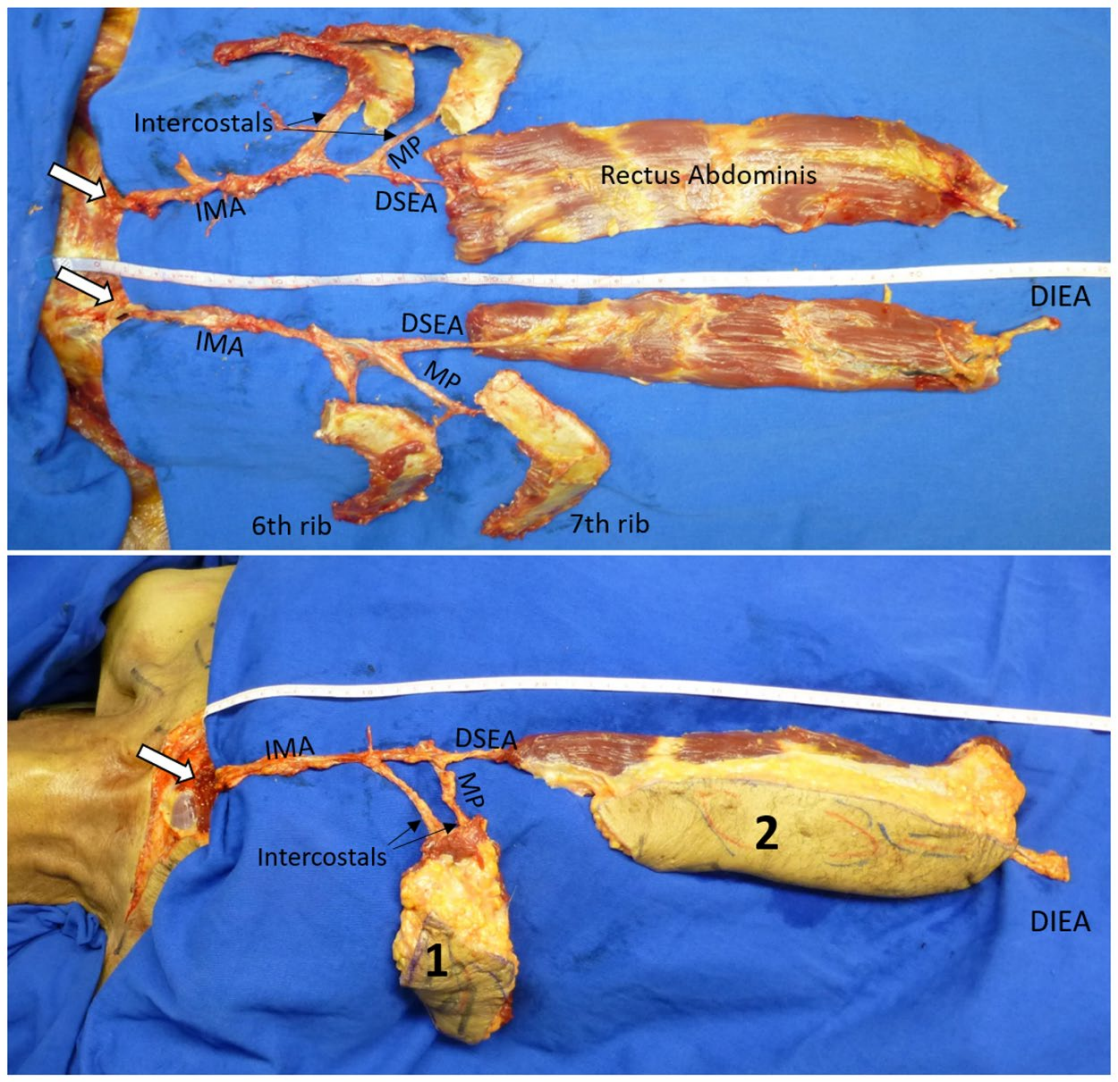
PIMOC cadaver dissection. (**A**) The IMA pedicle gives rise to the intercostal arteries and bifurcates distally into the musculophrenic (MP) artery and the deep superior epigastric artery (DSEA). The DSEA anastamoses with the DIEA after supplying the rectus abdominis. The 6^th^ and 7^th^ ribs are supplied by the MP and individual intercostal arteries. (**B**) The PIMOC with its IMA pedicle, osteomyocutaneous components including the 6^th^ and 7^th^ ribs (1), and myocutaneous vertical rectus abdominis component (2).

Rib dissection was completed from inside the pleural cavity. The final PIMOC composition included the IMA pedicle, the 6^th^ or 7^th^ rib with a slip of adjacent intercostal, serratus, or external oblique muscle, thoracic skin, and the VRAM (Fig. 1). The PIMOC was dissected en block and pivoted at the inferior border of the first rib to reach the head and neck region. Details of the PIMOC harvest in the cadaver is shown in Video, Supplemental Digital Content 1.

Variable normatives were tested with Kolmogorov-Smirnov. Quantitative variables were presented in minimum, maximum and mean with standard deviation. Categorical data were compared using Students’s t-test, Fisher’s exact test, and Mann-Whittney’s U-test, as appropriate. Values of *p* < 0.05 were considered significant.

### Clinical Cases

This study was approved by the Ethical Committee of the University of São Paulo Medical Center. All methods were performed in accordance with the relevant guidelines and regulations. From July 2012 to June 2014, we selected five frozen-neck male patients with ages ranging from 31 to 68 to undergo head and neck reconstruction utilizing the PIMOC. Patients were counseled on the surgical risks. Informed consent for the surgical procedure and for publication of identifying information/images was obtained. They underwent selective medical work-up for surgical risk stratification.

#### Inclusion criteria

- Vessel-depleted neck anatomy evidenced by computed tomography angiography (CTA);
- Radiation-induced tissue necrosis or fibrosis;
- Complex defect including mucosa, soft tissue, bone and skin;
- Scarce loco-regional or free flap options;

#### Exclusion criteria

- Adequate recipient neck vessels for reliable free tissue transfer;
- Suitable loco-regional flap options;
- Poor ventilatory status;
- Previous use of both internal mammary vessels for cardiac revascularization, or proven ischemic cardiac condition;
- Refusal of the terms and conditions for the proposed technique.

#### Surgical Technique

**Figure 2:**
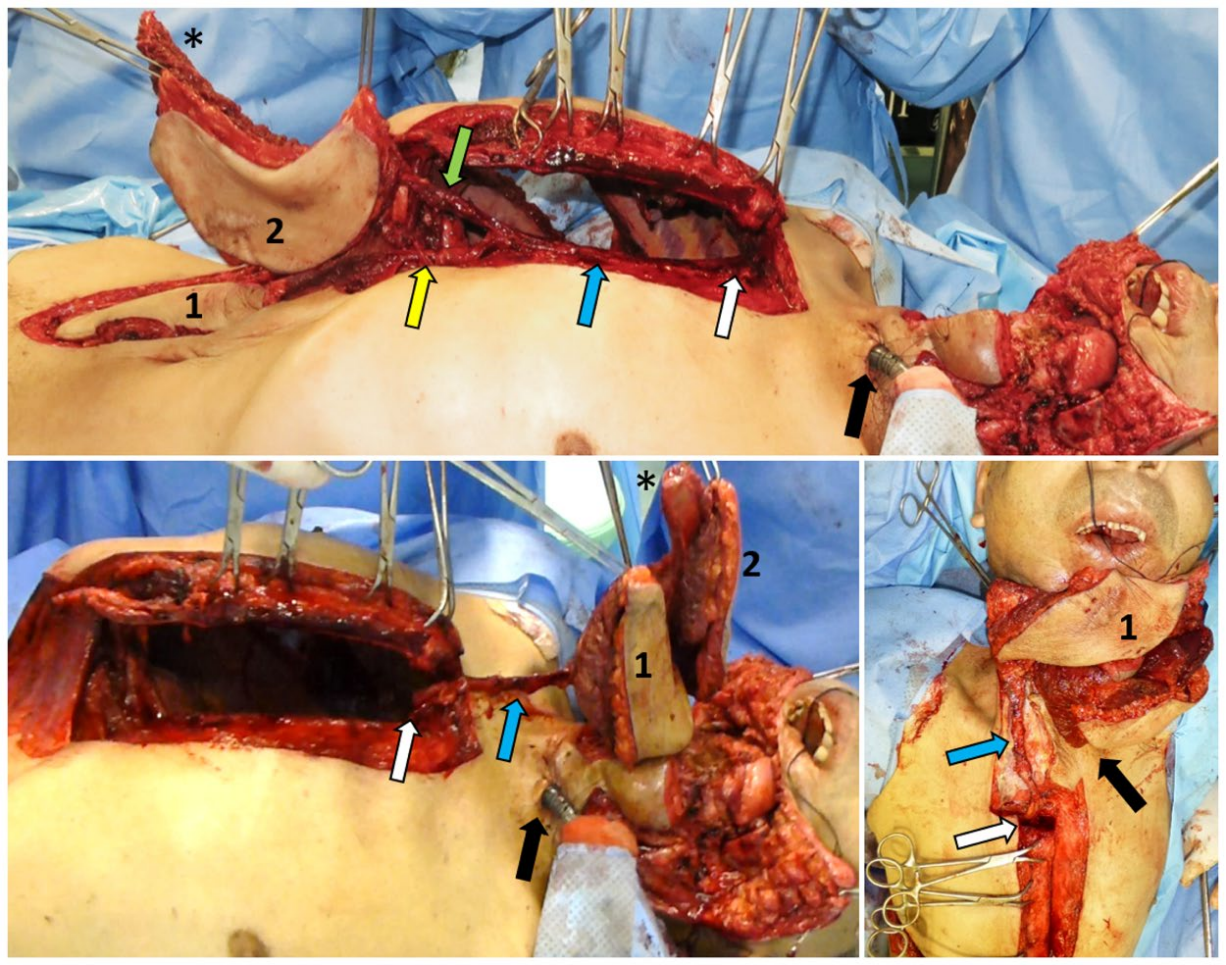
PIMOC surgical technique. (**A**) Exposure of the mediastinum through an inverted L sternotomy incision and dissection of the IMA pedicle (blue arrow). The VRAM (1) is supplied by the DSEA (yellow arrow). The osteomyocutaneous component (2) contains the 7^th^ rib (*) in this case and is supplied by both the musculophrenic and intercostal arteries (green arrow). (**B**) Rotation of the PIMOC cephalad. The pivot point of the IMA pedicle is at the lower margin of the first rib (white arrow). (**C**) Flap inset. The IMA pedicle positioned within the loose subcutaneous tissue at least 3 cm from the tracheostomy site (black arrow). The rib osteomyocutaneous component was used for reconstruction of the mandible and oral lining, and the VRAM component was used for external coverage.

Prior to PIMOC dissection, we explored the neck vessels viability and confirmed a lack of vessel patency suitable for safe microsurgical tissue transfer. The PIMOC was then harvested as described in the cadaver study, with one exception. In the cadaveric dissections, the rib cartilage was included in the flap to create a versatile chimeric design in order to expand our reconstructive options. In the clinical setting, costal cartilages were not included in the flap because the mandible defects required bony reconstruction only. Costal cartilage was therefore preserved to prevent undue donor site morbidity. The 6^th^ or 7^th^ rib osteomyocutaneous component was selected based on vessel caliber and ease of pedicle dissection. The internal mammary pedicle was pivoted at the inverted L osteotomy, rotated around the first rib and clavicle, and positioned within the loose subcutaneous tissue at least 3 cm away from the tracheostomy (Fig. 2). Final inset with bone fixation and multi-layered closure was performed.

The donor site was closed addressing the thoracic cavity and the abdomen separately. For thoracic cavity closure, polypropylene mesh was used to span the rib defect overlying the exposed pleural cavity. The mesh was sutured to the periosteum along the inferior border of the superior rib resection, the superior border of the inferior rib resection, and the medial and lateral remaining rib segments. A thoracic tube was placed in the pleural cavity and exited caudal to the rib resection, along the mid axillary line. Primary closure of the overlying anterior serratus and external oblique muscles, and skin was performed to cover the mesh. The inverted L sternotomy was securely repaired with wire fixation.

After harvest of the VRAM and the SSEP skin island based on pinch test, the abdominal defect was primarily closed by approximation of the overlying soft tissues. Polypropylene mesh was also used in the VRAM closure, where it was sutured on top of the retrorectus fascia, prior to skin closure around bulb-suction drains. Blood transfusions were performed as necessary to maintain a hemoglobin level above 9 g/dL.

#### Patient Follow-up

The patients were closely followed by a multidisciplinary team, which included plastic surgery, intensive care, psychology, dentistry, and speech therapy. A methylene blue swallow study was routinely performed on post-operative day 6 to assess suture leak. Flap viability was clinically assessed by skin color, temperature and capillary refill every three hours for the first 48 hours after surgery. Long-term follow-up included evaluation of flap necrosis, quality of wound healing and vessel perfusion through CTA after 6 months. Patients were fed a high caloric protein diet through a nasoenteral feeding tube or a gastrostomy until the patients were able to receive adequate hydration and nutrition orally. Patient satisfaction with final reconstruction, speech and swallow outcomes were subjectively measured based on the patient’s perceived quality of life before and after the surgery and were graded as poor, moderate, fair and good.

## Results

### Anatomic Study

After a pilot study in 10 cadavers to verify the anatomy and feasibility of the proposed technique, we were able to dissect the PIMOC on each side in all 35 cadavers.

**Figure 3:**
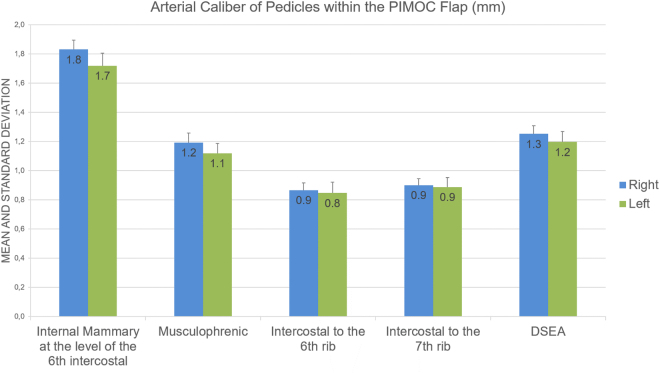
Arterial Caliber of Pedicles within the PIMOC. Mean values and standard deviation represented in millimeters.

Seventy flaps were bilaterally harvested in 27 (77%) male and 8 (23%) female cadavers. We observed that the anterior intercostal vessels arise from the internal mammary artery and the musculophrenic branch; the later typically giving rise to the intercostal vessels from the 6^th^ rib down. Arterial diameter and length of the internal mammary pedicle and its branches were not statistically different with regards to gender and laterality. Caliber of the IMA ranged from 1.5 to 2.4 mm; the internal mammary vein from 2.0 to 3.6 mm (Fig. 3). Caliber measurements of the smaller veins were excluded due to imprecision in cadaver specimens^21^. The superficial superior epigastric perforator (SSEP) was confirmed as the first perforator of the DSEA just after its division from the IMA at the inferior costal margin^19^. Pedicle length to the 6^th^ and 7^th^ rib osteomyocutaneous components ranged from 18.5 to 21.6 cm, providing adequate length for flap rotation to the face in all dissections. The VRAM component reached the head in all dissections.

### Clinical Cases

Prior to PIMOC surgery, patients were submitted to an average of 1.8 free flap and 2.7 loco-regional flap reconstructions, including the free fibula, pectoralis major, deltopectoralis, supraclavicular and tongue flaps. On average, each patient underwent 2.3 tumor resections and 5.4 previous surgeries (Table 1).

**Table 1:** Summary of our PIMOC Series.

Patient	Diagnosis	Stage	Background	Initial treatment	Previous recon-structions	Adju-vant Tx	Surgery performed	Defect	Recon-struction	Flap compo-sition	Complica-tions and treatment	LOS	Outcome	Follow-up
1. RSN M, 31 y	Cranial base pleomorph. sarcoma	Grade III	Malnutrition Gastrostomy	Extended field RTX + bilateral neck dissection	None	CTX RTX	Left partial mandibulo-maxillectomy, cranial base, nasal and oropharynx, skin resection	Communication between dura and nasal/oral cavity, cheek skin	Immediate L extended pedicle VRAM	VRAM	OCF: flap adv DS: no complications	45 days	Fair contour, fair oral intake and speech	11 mo death: tumor recurrence
2. RGS, M, 42 y	Anterior gingiva and mandible SCC	T4N1M0	Tobacco Alcohol Tuberculosis Gastrostomy	Anterior CMR with skin + bilateral neck dissection	Free ALT, Free LD, free IC, trapezius, SCM, Pectoralis major + mandibular plate	CTX RTX	Debridement previous flap + reconstruction plate removal	Angle to angle mandibulectomy, oral cavity, chin skin + oral incompetence	Delayed R PIMOC	6th rib, 2 skin islands: SSEP + VRAM	OCF: flap adv DS: no complications	28 days	Good contour, good oral intake and speech	12 mo death: tumor recurrence
3. CA, M, 54 y	Anterior gingiva and mandible SCC	T4N2cM0	Tobacco Alcohol Gastrostomy	Anterior CMR + bilateral neck dissection	Free fibula × 2, bilateral oral mucosa flap, SCM	CTX RTX	Reconstitution of oromandibular defect	Andy Gump deformity; angle to angle mandibulectomy, FOM, chin skin	Delayed R PIMOC	6th rib, 2 skin islands: IC perf + SSEP	Cervical skin dehiscence: flap adv DS: no complications	27 days	Good contour, good oral intake and speech	3 mo death: respiratory infection
4. VOG, M, 68 y	Left retromolar mandible SCC	T4N2aM1	Tobacco Alcohol Gastrostomy Gastrectomy from gastric ulcer	Left posterior CMR + bilateral neck dissection	Free fibula, free TFL, Pectoralis major, supra-clavicular, deltopectoralis, tongue flap	CTX RTX	Reconstitution of oromandibular defect	Left mandibulectomy, oropharynx, cheek skin + tongue adherence	Delayed R PIMOC	7th rib, 2 skin islands: IC perf + VRAM	Facial nerve neuropraxia OCF: flap adv DS sternal sinus: debride	25 days	Good contour, fair oral intake and speech	20 mo cranial base recurrence
5. CEO, M, 54 y	Tongue and FOM SCC	T4aN2bM0	Tobacco Alcohol Drug Abuse Gastrostomy	Anterior CMR + bilateral neck dissection	Free fibula × 2 pectoralis major supra-clavicular tongue flap, lip flap	CTX RTX	Reconstitution of oromandibular defect	Andy Gump deformity; angle to angle mandibulectomy, FOM, chin skin	Delayed R PIMOC	7th rib, 2 skin islands: IC perf + VRAM	Cervical skin dehiscence: flap adv DS: no complications	35 days	Good contour, fair oral intake and speech	14 mo speech therapy rehabilitation

M, male; SCC, squamous cell carcinoma; FOM, floor of mouth; CMR, composite mandible resection; ALT, anterolateral thigh flap; LD, latissimus dorsi flap; SCM, sternocleidomastoid flap; TFL, tensor fascia lata flap; CTX, chemotherapy; RTX, radiotherapy; SSEP, superficial superior epigastric perforator; VRAM, vertical rectus abdominis myocutaneous flap; IC, intercostal; OCF, orocutaneous fistula; DS, donor site; LOS, length of stay.

**Figure 4:**
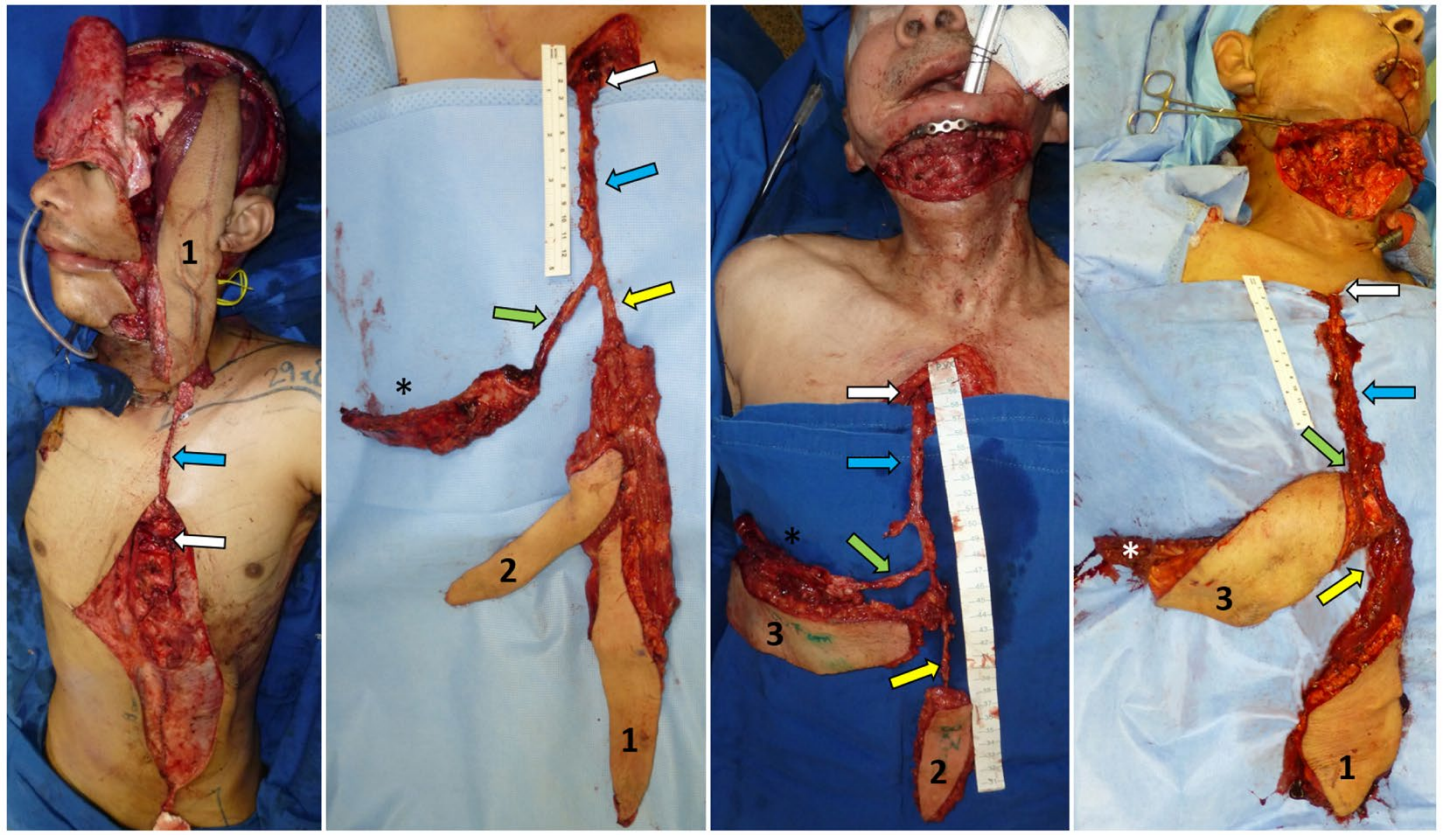
PIMOCs. (**A**) Patient 1. Left extended pedicle VRAM flap (1). (**B**) Patient 2. Right PIMOC with 6^th^ rib (*), SSEP (2) and VRAM (1). (**C**) Patient 3. Right PIMOC with 6^th^ rib osteomyocutaneous component (3) and SSEP (2). (**D**) Patient 5. Right PIMOC with 7^th^ rib (*) osteomyocutaneous component (3) and VRAM (1). Internal mammary artery (blue arrow); musculophrenic and intercostal arteries (green arrow); DSEA and SSEP arteries (yellow arrow).

**Figure 5:**
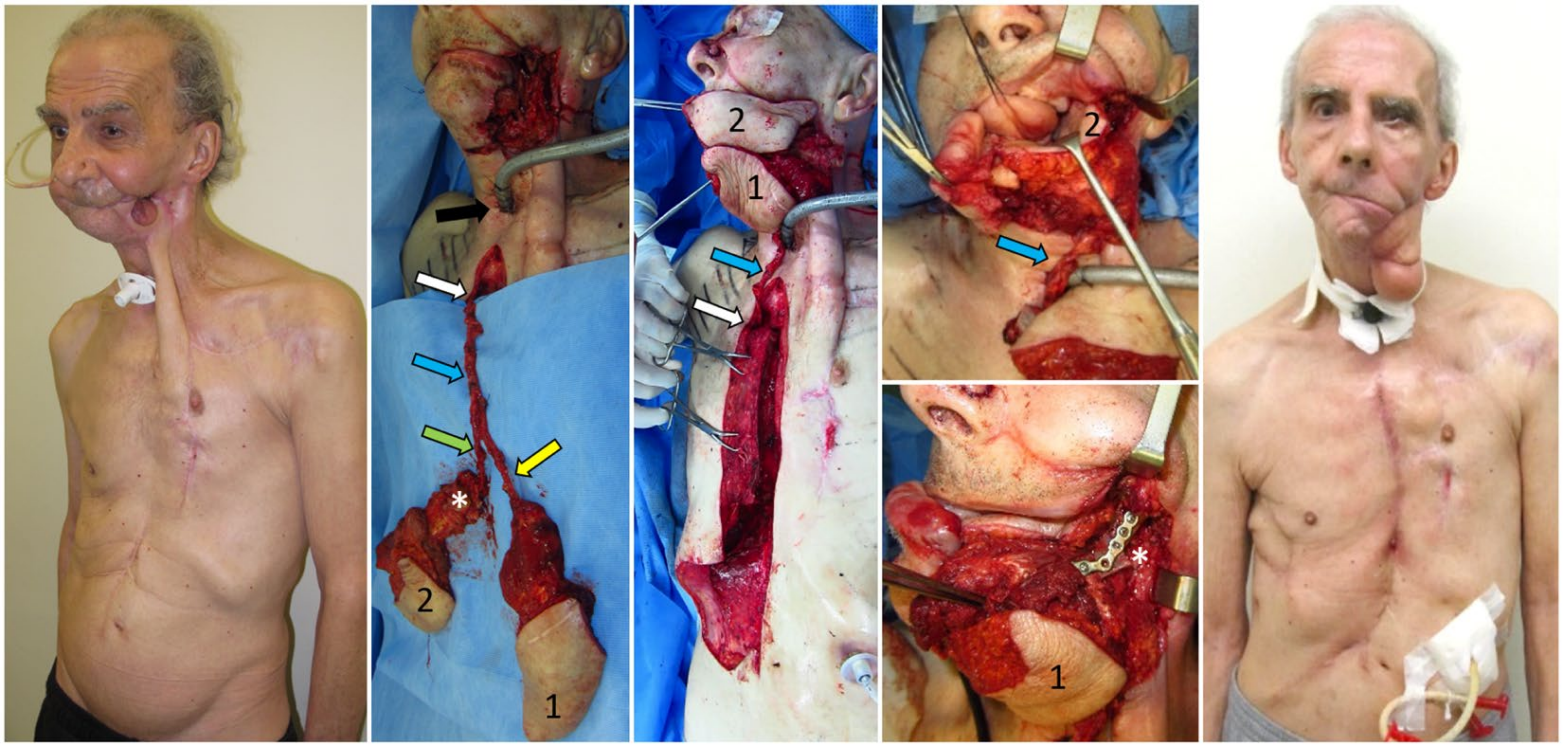
Patient 4 underwent composite mandibular resection for squamous cell carcinoma (SCC) of the mandible and floor of mouth with adjuvant chemotherapy and radiation therapy, followed by multiple failed reconstruction attempts. (**A**) Pre-operative full thickness defect involving the lateral cheek and hemi-mandible with an exposed, tethered tongue after failed pectoralis major, supraclavicular, deltopectoralis and tongue flap reconstructions. Note the laparotomy scar from gastrectomy secondary to gastric ulcer. (**B**) Harvest of the right PIMOC containing the 7th rib (*) osteomyocutaneous (2) and VRAM (1) components. (**C**) Rotation of the PIMOC to the contralateral face. (**D**) Inset of the intercostal perforator skin island for reconstruction of oral lining. (**E**) Fixation of the rib (*) for mandible reconstruction with large reconstruction plate. VRAM (1) for neck skin resurfacing. (**F**) Post-op 5 months with complete healing of the initial defect and donor site. The residual deltopectoralis flap was used for additional external coverage. The patient regained oral competence and speech and swallow capabilities. Planned revision of the excess external skin was postponed due to cranial base SCC recurrence. IMA (blue arrow); musculophrenic artery (green arrow); DSEA (yellow arrow); pivot point of IMA pedicle (white arrow); tracheostomy site (black arrow).

The PIMOC included a 6^th^ rib segment in two patients and a 7^th^ rib segment in two patients, three of which contained a myocutaneous component supplied by intercostal perforators. In four patients, we harvested the rectus abdominis muscle with overlying skin supplied by epigastric artery perforators, and, in two patients (patients 2 and 3), we included an SSEP cutaneous flap (Figs 4 and 5). In Video, Supplemental Digital Content 2, we demonstrate the dissection and clinical application of the PIMOC in salvage head and neck reconstruction cases.

All patients recovered in the Intensive Care Unit for an average of 4 days. Thoracic tubes were maintained post-operatively for an average of 3.6 days and were well tolerated by all patients. In all cases, inverted L sternotomy and the resection of a single rib did not compromise the ventilatory status of the patient. In one case, there was mild venous congestion of the flap that resolved after suture release over the pedicle (patient 5).

**Figure 6:**
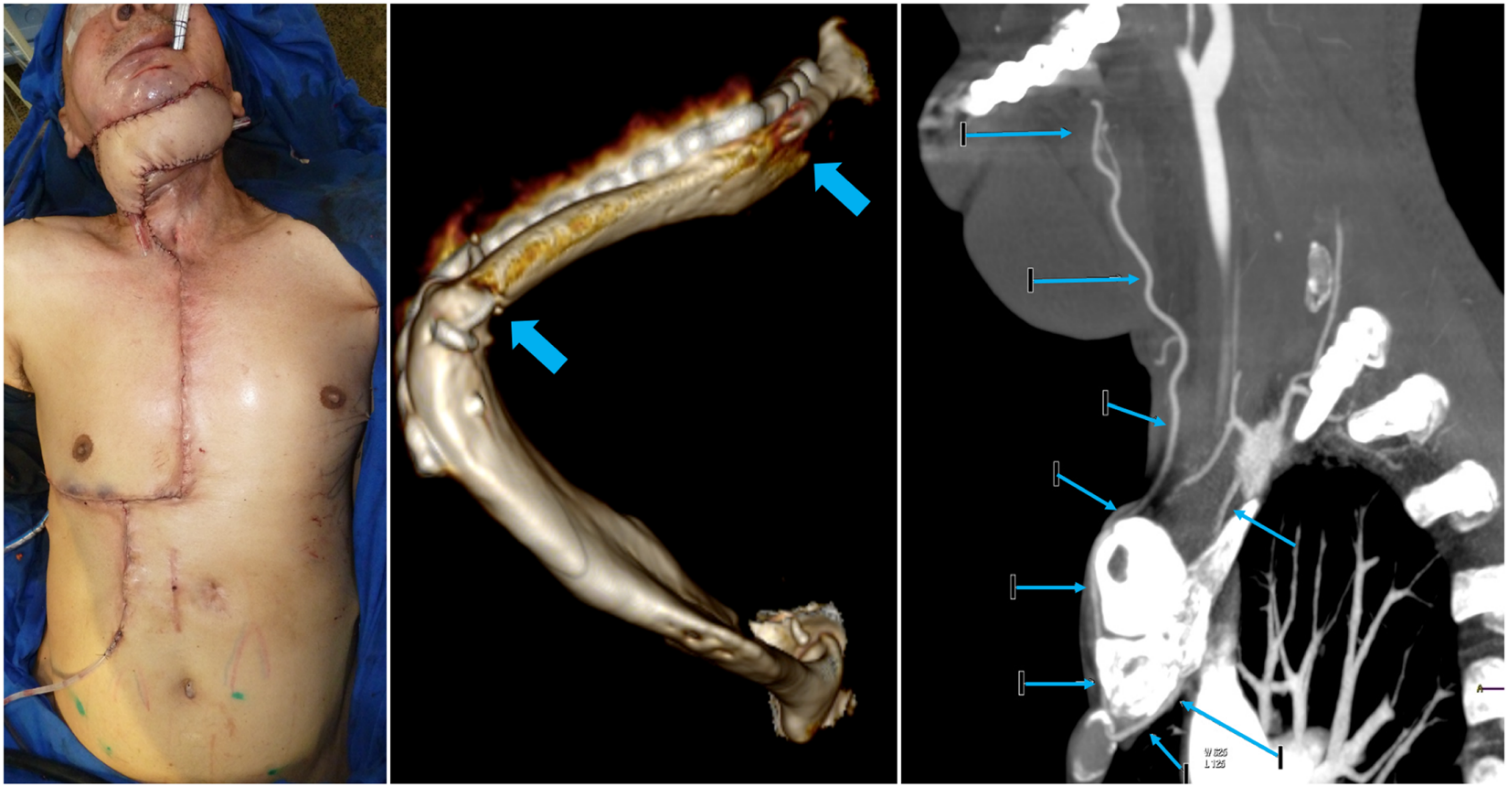
Post-op clinical cases. (**A**) Primary closure of the PIMOC donor site in patient 3. (**B**) 3D reconstruction of computed topography angiography (CTA) demonstrating osseous integration of the left mandibular body with the 7^th^ rib (blue arrows) in patient 4. There is increased ossification at 6 months post-op. (**C**) Sagital CTA demonstrating the internal mammary artery pedicle pivoted anteriorly around the first rib (**A**) and clavicle (**B**) and ascending into the head and neck region (blue arrows).

Donor sites were closed primarily in all cases without major donor site complications including respiratory compromise or sternal nonunion (Fig. 6A). There was one small sternal sinus that resolved with sternal wire removal and re-closure. All osseous segments remained viable and underwent adequate osseous integration, confirmed by post-operative CTA (Fig. 6B). Head and neck tissue fibrosis was replaced by healthy donor tissue in all cases. All patients experienced minor wound dehiscences and small salivary fistulae that resolved on average within 21 days with dressing changes or debridement and flap advancement. There were no complete flap losses or segmental flap necrosis. Hospital length of stay averaged 32 days and mean follow up was 12 months. All patients were satisfied with their final reconstruction result and gradually recovered oral competence with fair (1) or good (4) speech and swallow outcomes (Table 1).

## Discussion

Ariyan *et al*. and Hendel *et al*. observed that the caliber and arterial flow of anterior and posterior intercostal vessels are similar, confronting the belief of a posterior predominance at that time^22,23^. Their findings support our flap design, which includes rib segments supplied by the anterior intercostal artery. Furthermore, there is evidence that the vascular pedicle supplying the rib comes from both the superior and inferior borders^24^, which were included in our PIMOC. This vascularized bone graft is slightly convex and is amenable to multiple osteotomies and fixation sites, promoting better restoration of mandibular defects. The overlying skin and muscle may also be included to create an osteomyocutaneous flap through preservation of the intercostal perforators supplying the intercostal, anterior serratus, or external oblique muscles.

IMA caliber discrepancy, with right-sided vessels reportedly larger than the left, and variation in the presence and number of the venae comitantes has been described^19,25–27^. However, in our 70 cadaveric flap dissections, there was no difference in the IMA caliber with respect to gender or laterality (p < 0.005), and there was at least one vena comitans accompanying all arterial pedicles. This difference may be due to anatomical variations between the cadaver and live patient.

PIMOC dissection and isolation of the individual pedicles was reproducible bilaterally in all cadavers. The intercostal branches supplying the 6^th^ and 7^th^ ribs were consistent in length and were dissected in continuity with the rectus abdominis pedicle in all specimens. This increases flap versatility as flap design can be selected based on ease of dissection.

The VRAM component is a simpler dissection and provides a very wide arc of rotation to reach the occiput. The presence of the DIEA at its inferior border allows for anastomosis of an additional free flap or for supercharging the PIMOC (Fig. 1).

The absence of healthy and reliable recipient vessels in head and neck reconstruction is a significant and more frequently encountered problem. Many strategies have been described to improve recipient vessel availability in these cases. Cephalic vein transfer can be a reliable option and may be combined with the radial forearm flap^3,8^. The thoracoacromial vessels can also be used as recipients for microsurgical anastomosis, even after pectoralis myocutaneous flap harvest^8,28^. If available, the contralateral neck vessels may also suffice; however, a vein graft or AV loop is often necessary, which requires additional vessel anastomoses and, as such, increased risk of complications^3,4^.

The internal mammary vessels have long been used for microsurgical mammary and chest wall reconstruction^29–31^. They are, however, less commonly utilized for head and neck reconstruction. Urken *et al*. and Urban *et al*. described a series of reconstructions with an internal mammary pedicle, accessed through the third intercostal space, as a recipient for a free rectus abdominis muscle flap for salvage head and neck cases^14,32^.

The internal mammary vessels have also been used for pedicled flaps. In 1971, Strauch described a complex experimental IMA pedicled flap in a canine model. His dissection extended through a sternotomy incision to elevate an osteomyocutaneous rib flap that was rotated to the cephalic region^33^. A few plastic surgeons applied a similar technique in the clinical setting; however, the approach was abandoned due to technical complexity, lack of clear indications, anatomic variations, and donor site morbidity^26,34–36^. Ketchum *et al*. and Cook *et al*. transferred pedicled 7^th^ and 5^th^ rib osseous-only flaps, respectively, via sternotomy incisions and fracturing of the adjacent synchondroses for mandibular reconstruction^34,35^. Later, Arons and Guyron described a rib osteomyocutaneous chimeric pedicle flap harvested through cartilaginous rib resections that was rotated to the lower third of the face in a patient with calcinosis universalis who underwent 29 previous surgeries including two free fibulas. The flap was delayed for 8 days due to flap congestion^36^.

Based on these challenges and our previous anatomic studies, we sought to create a novel chimeric flap for salvage head and neck reconstruction and were able to establish a reliable and reproducible harvesting technique for what we named the PIMOC. This procedure was successfully applied to five clinical cases of salvage head and neck reconstruction with satisfactory subjective patient outcomes.

The osteomyocutaneous rib flap proposed by Strauch *et al*. was discouraged and limited in its full development due to dissection difficulties and donor site morbidity. Their approach to the IMA in the canine model created an unstable thorax, resulting in 70% mortality and 100% severe thoracic complications^33^. Ketchum *et al*. performed a median sternotomy incision and fractured all synchondroses from the 1^st^ to 6^th^ ribs in order to access the 7^th^ rib flap, resulting in thoracic instability in the live patient^34^. Others accessed the internal mammary vasculature through conventional sternotomy incisions with cartilaginous rib resections to perform pedicled rib flaps which also resulted in thoracic instability^35,36^. Many of the patients who would benefit from a procedure such as the PIMOC are long-time smokers and do not have the functional ventilatory reserve to tolerate such instability.

To limit donor site insult, we performed an inverted L sternotomy with preservation of the manubrium as described in the cardiothoracic literature^37^, and maintained an intact skin island separating the sternotomy incision from the tracheostomy site. The inverted L sternotomy has less donor site morbidity than the average 3% seen with the standard total median sternotomy^37–40^. The transverse osteotomy at the first intercostal space preserves the structure and function of the thoracic cavity, allowing adequate post-operative consolidation of the sternum. This approach minimizes contamination from the tracheostomy site and consequent mediastinal infection (Fig. 2). Prevention of pneumothorax was managed with a thoracic tube to allow adequate postoperative lung expansion and preclude pleural effusion. Although there is risk for sternal bone nonunion, we achieved adequate sternal wire fixation and bony union, and observed only one draining sinus, which resolved with excision of the involved sternal wire and reclosure. A thoracoscopic approach may also facilitate the dissection and reduce the physiologic burden to the donor site. This method is currently being studied by our group.

The rib has been criticized for providing a weak bone substitute for a functional mandible reconstruction. However, the availability of the rib as a vascularized bone graft with its independent blood supply is an important alternative for the reconstructive surgery. The long-term strength of the rib in mandibular reconstruction has yet to be determined. It will likely require further bone hypertrophy before supporting dental implantation, and it remains unclear if it would actually be possible in such cases. However, ossification and bone width expansion of vascularized rib grafts have been shown in humeral and vertebral reconstructions^41,42^. Although not quantified, we see this trend in our patients as well (Fig. 6B). Moreover, ribs 6 and 7 were selected based on increased bone width in comparison to the other ribs. This technique provides a long pedicle to a vascularized rib graft that may also be used as a free flap for other bony reconstructions throughout the body.

Prior to the development of the PIMOC technique, our customary procedure of choice for salvage head and neck reconstruction was mobilization of distant recipient vessels for free tissue transfer or regional flap advancement with suboptimal results. In fact, the first patient to enroll in the PIMOC flap protocol (patient 1) had a preoperative CT angiogram demonstrating a lack of reliable recipient neck vessels for safe free flap transfer. This patient was overall quite thin with the majority of his available redundant tissue in the abdomen. Instead of mobilizing the internal mammary vessels for free flap anastomosis^14,32^, we favored a pedicled flap to include the extended VRAM for the reconstruction in this scenario.

Access to the internal mammary pedicle is directly within the mediastinum on the undersurface of the ribs. If the surgeon is less comfortable performing sternotomy incisions, a multidisciplinary approach with a cardiothoracic surgeon can be utilized. Dissection of the vascular pedicle is tedious; however, it is possible to isolate both osteomyocutaneous and myocutaneous components bilaterally. The flap can also be inset into the contralateral face (patient 4). The decision to pivot the flap over the first rib resulted in pedicle shortening by approximately 6 cm; however, this did not compromise flap reach and instead avoided undue subclavicular dissection (Fig. 6C). One may argue that mediastinal access with adjacent neck exposure may increase mediastinal infection risk from salivary fistulae; however, no salivary fistulae in continuity with the mediastinum or thoracic infections were noted in our series.

All patients presented with minor wound dehiscences, primarily due to poor local tissue quality of the recipient site as a result of scar, fibrosis, friable inflammatory tissue, radiation damage, multiple surgeries, previous infections, and salivary contamination. The skin paddles from the PIMOC flap supplied healthy tissue with a robust blood supply and presented no partial or total necrosis. Flap inset was performed in a tension-free manner, with the vascular pedicle loosely positioned within the subcutaneous tissue of the neck. While our reconstructive approach focused on resurfacing as much damaged tissue as possible – removing scared, fibrotic, poorly vascularized tissue in order to substitute it with new soft, malleable, well-vascularized flap tissue – we also understood that it is impossible to resect all damaged tissue in the region, and therefore planned for some expected peripheral wound breakdown. The flap inset was designed accordingly to protect the vascular pedicle and main flap vessels from salivary fistula, so that if a fistula were to present peri-operatively, it would be in an area of the neck that was manageable. Improvement in wound dehiscence rates may be achieved through more selective radiotherapy regimens, improved patient nutrition, and optimized blood pressure and tissue oxygenation.

The PIMOC is an additional resource for the astute reconstructive microsurgeon and has several valuable characteristics. It is a pedicled composite flap with malleable individual tissue components that easily extends to the head and neck region, allowing three-dimensional functional reconstruction without the need for microsurgical anastomosis in an unfavorable neck. Long individual vascular pedicles within the flap increase versatility of each flap component. The main blood supply is based off of the well-known internal mammary artery vascular system, which has been utilized for cardiac revascularization for over 30 years^43,44^. The flap components are well-studied, such as the rectus abdominis, which is regularly used for breast and perineal reconstruction, and rib grafts for craniofacial surgery^45–47^. The distant donor site allows for a simultaneous multi-team approach and provides non-irradiated tissue with tolerable morbidity.

## Conclusion

The cadaveric anatomic study was essential for the development of a viable PIMOC flap. The PIMOC provides well-vascularized chimeric tissue with variable composition and individualized components for optimal salvage head and neck reconstruction. Although technically complex, flap dissection is reproducible and reliable. The inverted L sternotomy and harvest of the 6^th^ or 7^th^ rib with rectus abdominis muscle render an acceptable donor site. PIMOC reconstruction improved functional and structural outcomes and enhanced the quality of life for tertiary head and neck cancer patients.

### Data availability

The datasets generated during and/or analysed during the current study are available from the corresponding author on reasonable request.

## Electronic supplementary material

Supplementary Video Legends

Supplementary Video 1

Supplementary Video 2
