# Supplementary material for: A New Pedicled Internal Mammary Osteomyocutaneous Chimeric Flap (PIMOC) for Salvage Head and Neck Reconstruction: Anatomic Study and Clinical Application
Source: Sci Rep. 2017 Oct 11;7:12960. doi: 10.1038/s41598-017-13428-7 (PMC5636798; doi:10.1038/s41598-017-13428-7)
Supplement: Supplementary file 1 — Supplementary Video Legends [file 41598_2017_13428_MOESM1_ESM.pdf]

A New Pedicled Internal Mammary Osteomyocutaneous Chimeric Flap (PIMOC) for Salvage Head and Neck Reconstruction: Anatomic Study and Clinical Application

Guilherme C **Barreiro**, MD, PhD[1,*]; Chelsea C **Snider**, MD[2]; Flavio H F **Galvão**, MD, PhD[3,+]; Rachel R **Baptista**, MD[1,+]; Kiril E **Kasai**, MD[1,+]; Daniel M **dos Anjos**, MD[1,+], Marcus C. **Ferreira**, MD, PhD[4,+]

VIDEO LEGENDS

Video, Supplemental Digital Content 1. Demonstration of PIMOC harvest in the cadaver.

Video, Supplemental Digital Content 2. Demonstration of the clinical application of the PIMOC for salvage head and neck reconstruction.
